# The Distinction between Incomplete Fracture of the Neck of the Femur and Separation of the Epiphysis

**Published:** 1904-11-05

**Authors:** 


### THE DISTINCTION BETWEEN INCOMPLETE FRACTURE OF THE NECK OF THE FEMUR AND SEPARATION OF THE EPIPHYSIS.

In a very lucid paper appearing in the *Medical News* of September 24, Dr. Royal Whitman describes the salient points by which the above injuries may be distinguished, and urges the necessity of an exact discrimination of the site of injury in order that the subsequent treatment may be correct. He states that fracture of the neck of the femur is relatively common in early life, and that epiphyseal separation, when it does occur, is most frequent in adolescence, and comparatively rare in the earlier years of life. He points out that it is difficult to produce separation of the epiphysis experimentally, even when the force is applied with this purpose. If, therefore, epiphyseal separation is, as some have thought, relatively common at the hip joint there must be some local anatomical or pathological predisposition. Anatomically, the predisposition is in favour of fracture of the neck, because that is where the bone is thinnest; and when fracture is accidentally produced during the reduction of congenital displacement the lesion occurs through the neck of the bone. An epiphyseal junction is not a weak point in the bone during childhood except as the effect of local or general disease. It is, however, relatively weak in adolescence, and it is at this period of life that separation of the femoral epiphysis is least rare. Both in fracture and separation of the epiphysis the lesion is usually incomplete.

### Classification.

Dr. Whitman includes three classes of cases: (1) Simple, direct fracture of the neck, usually incomplete, occasionally complete. (2) Direct epiphyseal disjunction, usually incomplete, in rare instances complete. (3) Epiphyseal coxa vara: an indefinite class in which the deformity is at or in close proximity to the epiphyseal junction; the patients are of a type in which the so-called static deformities of adolescence are common, and injury may be a predisposing, aggravating, or direct cause of the distortion.

### Symptoms.

Fracture of the neck of the femur is usually the result of direct violence, such as falling from a height. The disability is immediate and lessens as repair proceeds, and in a few months the patient may be practically well, except for a slight limp and limitation of motion dependent upon the depression of the neck of the femur (traumatic coxa vara).

Epiphyseal disjunction is usually a more gradual process. The patients are almost always adolescents, very often overgrown and overweighted. The injury may be slight, so slight perhaps as to pass unnoticed. The first symptoms are a limp, a little stiffness, and discomfort referred to the hip and thigh. These symptoms may persist for weeks or months when, usually after over-exertion or another injury, the disability may suddenly increase; an indication of a more extensive secondary fracture.

### Physical Signs.

In incomplete fracture there are shortening and outward rotation of the limb with elevation and prominence of the trochanter. Movement may be practically free, except in abduction, which is much restricted, particularly when the limb is flexed. In partial separation of the epiphysis the outward rotation of the limb is more marked, but the shortening is less, usually not more than half an inch. As a rule the limb is completely extended and in many instances a resistant swelling is apparent in Scarpa's space. The trochanter is not so prominent, but movement is much more limited than in fracture. In many instances it is completely prevented by muscular spasm, and there may be effusion within the joint and tenderness on pressure.

The difference between the diagnostic features in the two classes of cases is entirely due to the situation of the injury, and this, in many instances, may be determined by means of the $x$-rays.

It may be concluded that when fracture at the hip-joint in a child follows a fall from a height, or other considerable violence, the injury, as in older patients, is usually of the middle or outer portion of the neck. In adolescence, on the other hand, when progressive disability follows slight injury, and when the physical signs are similar to those described above, separation of the epiphysis may be suspected.

### Treatment.

The object of treatment should be the restoration of normal form rather than palliation of the symptoms; and the method employed will differ according to the time that has elapsed from the injury and the exact nature of the lesion—whether fracture or separation of the epiphysis.

If, in the case of incomplete fracture, the patient be seen immediately after the injury, and if there is but very slight deformity, rest in bed until complete consolidation has taken place will ensure a cure. But if the injury be unrecognised, as is frequently the case, and the patient is allowed to walk about, the weakened bone will give way under the weight of the body, and the deformity and disability will progressively increase.

If the patient comes under treatment before consolidation is completed, the normal angle between the neck and shaft of the bone may be restored by forcibly abducting the thigh and utilising the upper border of the acetabulum as a fulcrum. If the limb be then fixed for a sufficient time in a plaster of Paris splint at the limit of normal abduction, anatomical and functional cure may be assured. Or, if consolidation be complete before treatment is commenced, proper elevation of the neck may be restored, as in simple coxa vara, by the removal of a wedge of bone at the base of the trochanter. In the case of partial separation of the epiphysis a very slight displacement is sufficient to greatly impair the function of the joint. Moreover the displacement is usually so gradual that it is accompanied by repair, and although the weakening may be enough to allow of progressive deformity, yet the amount of repair is usually sufficient to prevent rectification by forcible abduction. Direct operative intervention is therefore the treatment of selection, unless the displacement is so slight that it does not much interfere with the function of the joint. The operator should direct his endeavour to the exact replacement of the head of the bone, or, in the slighter cases, to the removal of the angle formed by the inner extremity of the neck, which interferes directly with flexion.

Dr. Whitman draws a distinction between the deformity due to displacement of the epiphysis and epiphyseal coxa vara. The latter condition most often commences in adolescence; it is due to gradual yielding of the bone on the outer side of the epiphysis, and as the deformity is curved instead of angular, treatment by operation is not so satisfactory as in the case of separated epiphysis.

